# Measurable Cytokine Concentrations in Pig Seminal Plasma Are Modified by Semen Handling and Storage

**DOI:** 10.3390/biology9090276

**Published:** 2020-09-07

**Authors:** Lorena Padilla, Isabel Barranco, Inmaculada Parrilla, Xiomara Lucas, Heriberto Rodriguez-Martinez, Jordi Roca

**Affiliations:** 1 Department of Medicine and Animal Surgery, Veterinary Science, University of Murcia, 30100 Murcia, Spain; lorenaconcepcion.padilla@um.es (L.P.); isabel.barranco@udg.edu (I.B.); parrilla@um.es (I.P.); xiolucas@um.es (X.L.); 2 IMIB-Arrixaca, Regional Campus of International Excellence, Campus Mare Nostrum, University of Murcia, 30100 Murcia, Spain; 3 Department of Biology, Faculty of Sciences, University of Girona, 17003 Girona, Spain; 4 Department of Biomedical and Clinical Sciences (BKV), Linköping University, SE-58185 Linköping, Sweden; heriberto.rodriguez-martinez@liu.se

**Keywords:** seminal plasma, storage, cytokines, pig

## Abstract

Sample handling and storing are critical steps for the reliable measurement of circulating biomolecules in biological fluids. This study evaluates how cytokine measurements in pig seminal plasma (SP) vary depending on semen handling and SP storage. Thirteen cytokines (GM-CSF, IFNγ, IL-1α, IL-1β, IL-1ra, IL-2, IL-4, IL-6, IL-8, IL-10, IL-12, IL-18 and TNFα) were measured using Luminex xMAP^®^ technology in individual seminal plasma (SP) samples (*n* = 62) from healthy breeding boars. Three separate experiments explored the delay (2 h and 24 h) in SP collection after ejaculation (Experiment 1) and SP storage, either short-term (5 °C, −20 °C and −80 °C for 72 h, Experiment 2) or long-term (at −20 °C and −80 °C for two months, Experiment 3), before analysis. Levels in fresh SP-samples were used as baseline control values. Delays in SP harvesting of up to 24 h did not substantially impact SP cytokine measurements. Some cytokines showed instability in stored SP samples, mainly in long-term storage. Ideally, cytokines in pig SP should be measured in fresh samples harvested within 24 h after ejaculation. If storage of SP is imperative, storage conditions should be adjusted for each cytokine.

## 1. Introduction

Seminal plasma (SP) is a complex fluid, with biomolecules playing key roles in sperm function, fertilization and even embryo development and implantation [1,2]. Indeed, SP components are directly involved in essential functions of the spermatozoa, such as motility and capacitation [3,4]. In addition, SP components are also involved in the regulation of the uterine immune environment facilitating the development and implantation of embryos [5]. Consequently, SP components are currently being explored for potential biomarkers of male (in)fertility [6,7]. Among these biomolecules are cytokines, low-molecular-weight signaling proteins/peptides involved in intercellular communication, tissue homeostasis and the body’s immune response [8,9,10]. Male reproductive organs, including the testes, epididymides and accessory sex glands, synthesize cytokines, with a variable amount released into the SP [11]. Functional changes in the male reproductive organs lead to differences in cytokine synthesis, therefore, differences in SP cytokine levels are mirrored in reproductive (dys)function [12,13,14]. For instance, some SP cytokines were postulated as biomarkers of varicocele (interleukin (IL)-37 [15]), prostatitis (IL-8 [16,17]) and unspecific male infertility (panel with eight cytokines [18]). Moreover, SP cytokines are involved in pregnancy success, since they aid modulation of the immune response of female reproductive tissues to spermatozoa and embryos [19,20]. Therefore, accurate quantification of circulating SP cytokine levels could be of practical use for the clinical diagnosis of both male reproductive dysfunction and fertility.

However, since sample handling and storage are crucial steps, data resulting from improper handling of biological fluids as SP may not reflect true values, leading to unreliable diagnoses. Accordingly, several studies, mainly in humans, were conducted to define handling and storage guidelines of blood samples for cytokine measurements. For instance, these guidelines proposed separating the blood plasma as soon as possible (within one hour) and storing the blood plasma samples at −80 °C for no more than two years [21,22,23,24]. However, such studies have not yet been performed for semen samples. Semen is a body fluid different to blood, both in cellular and plasma composition, therefore, appropriate guidelines for biomolecules quantification may differ. This study aims to evaluate the influence of semen handling and SP storage on SP cytokine measurements, with a particular interest in breeding male pigs. To accomplish this aim, three experiments, simulating realistic scenarios, were carried out with seminal samples from breeding male pigs, a major livestock species [25] and a highly appreciated animal model for human medicine [26,27].

## 2. Materials and Methods

### 2.1. Animals, Ejaculates and Seminal Plasma Collection

Semen donors were housed and managed in accordance with the European Union rules for animal welfare. The experiments were approved by the Bioethics Committee of the University of Murcia (research code: 639/2012).

Healthy and fertile mature Pietrain boars housed in individual pens in environmentally controlled (15–25 °C and 16 h of light per day) barns of a commercial artificial insemination (AI) center (Calasparra, Topigs-Norsvin Spain) were randomly chosen as ejaculate providers. The boars were provided water ad libitum and were fed commercial feedstuff formulated to meet the nutritional requirements of AI boars. The boars underwent regular collection of two ejaculates per week to prepare liquid semen AI doses. Entire ejaculates were collected using Collectis^®^, a commercial semiautomatic equipment (IMV technologies, L’Aigle, France). Semen aliquots used in the experiments came from ejaculates that fulfilled the quantity and sperm quality requirements for the elaboration of liquid semen AI doses [28], including bacterial contamination under 300 × 10^6^ colony-forming units/mL. SP was harvested after double centrifugation (Rotofix 32A; Hettich Zentrifugen, Tuttlingen, Germany) of the semen (1500× *g*, for 10 min at room temperature, RT). The second supernatant, once microscopically verified to be cell-free, was handled as indicated in each experiment (see Section 2.3 experimental design).

### 2.2. Cytokine Measurements in Seminal Plasma Samples

The concentrations of thirteen cytokines were measured in undiluted SP samples using Luminex xMAP^®^ technology and a multiplex assay, MILLIPLEX^®^ MAP kit Porcine Cytokine/Chemokine Magnetic Bead Panel (Cat#PCYTMG-23K-13PX for pig reactivity, Merck Millipore, Burlington, MA, USA), following the protocol described by the manufacturer for plates with 96 wells. The thirteen quantified cytokines/chemokines were granulocyte macrophage colony-stimulating factor (GM-CSF), interferon-gamma (IFNγ), IL-1α, IL-1β, IL-1ra, IL-2, IL-4, IL-6, IL-8, IL-10, IL-12, IL-18 and tumor necrosis factor-α (TNFα). Standard seven-point curves for each cytokine were generated. A serum matrix and controls, both provided in the kit, were used to ensure accurate measurements of cytokine SP-concentrations. Aliquots of 25 µL of each standard and control were added into the appropriate wells and 25 µL of assay buffer was added into the sample and blank wells. Afterward, 25 µL of serum matrix was added into the blank, standard and control wells and 25 µL of undiluted SP was added into the sample wells. Once sonicated and vortexed, 25 μL of bead solution was added to each well. The plates were then incubated with continuous shaking at 4 °C in a dark chamber overnight (16 h). In the morning, the wells were emptied and washed three times with the washing solution provided in the kit. Then, 50 μL of detection antibodies were added to each well and the plates were incubated for 120 min at RT in a dark chamber. Next, 50 µL of streptavidin–phycoerythrin was added to the wells and the plates were incubated again for 30 min. After incubation, the wells were washed three times with the washing solution and 100 µL of sheath fluid was added to each well. The plates were run on a MAGPIX^®^ analyzer (Luminexcorp, Austin, TX, USA). The xPONENT version 4.2 (Luminexcorp) and MILLIPLEX^®^ Analyst Version 5.1 (Merck Millipore) were used for acquisition and data analysis, respectively. The median fluorescence intensity, analyzed using a 5-parameter logistic curve fit, was used to calculate cytokine concentrations, which were expressed in pg/mL. A minimum of two technical replicates of each SP sample were used for each seminal cytokine quantification. The final concentration recorded was the mean of the concentrations measured in the technical replicates. To normalize data from multiple plates, a single standard curve was created using the standard curve from each plate. The assays showed <10% intra-assay and <15% inter-assay coefficient variations. The technical replicates showed good reliability, considering the intraclass correlation coefficients were >0.8 [29].

### 2.3. Experimental Design

Three separate experiments were conducted with the ultimate goal of defining practical guidelines for semen and SP handling, including storage when attempting reliable cytokine measurements.

#### 2.3.1. Experiment 1: Interval Ejaculation–Seminal Plasma Harvesting

In most cases, and normally for working, technical or time reasons, SP cannot be harvested immediately after ejaculation, with semen samples remaining unprocessed for variable periods or even being sent unprocessed to clinical laboratories. Based on this reality, this experiment aimed to evaluate the effects of delaying the harvesting of SP for 2 h or even up to 24 h to mimic the above-listed possible events. Individual semen samples from twenty ejaculates (one per boar) were each split into three aliquots immediately after ejaculation. The SP from each aliquot set was harvested at different intervals, namely immediately after ejaculation (baseline samples) or after 2 h or 24 h of storage at 17 °C, which is the customary temperature maintained for liquid, extended pig semen. The resulting SP samples were kept frozen at −80 °C until cytokines were measured. This freezing storage temperature was chosen because it was recommended for the measurement of circulating body fluid cytokines in clinical trials with multiplex immunoassays [21]. The SP samples were stored over 6 months and thereafter thawed at RT for 60 min before analysis.

#### 2.3.2. Experiment 2: Storage and Transport Conditions for Seminal Plasma before Analysis

Boar semen production centers are, for sanitary reasons, located in remote places away from clinical laboratories. SP samples must therefore be shipped to a central laboratory for cytokine measurements and, consequently, must be stored for variable times until analysis, like human SP samples [30,31]. Accordingly, this second experiment evaluated realistic procedures for shipping and/or storage of SP samples. Individual SP samples were harvested from 22 ejaculates immediately after ejaculation (one sample per ejaculate and boar), and each was split into four aliquots. The first set of aliquots was kept at RT and processed for cytokine measurement within the first 4 h after SP harvesting (baseline samples). The other three sets of aliquots were stored, one chilled (5 °C; Zanussi Tropic System, Electrolux España S.A.U, Madrid, Spain) and the other two frozen, at either −20 °C (Zanussi Tropic System) or −80 °C (Ultra-Low Freezer; Haier, Qingdao, China). The SP samples were stored for 72 h and thereafter warmed at RT for 60 min before cytokine measurement.

#### 2.3.3. Experiment 3: Effects of Frozen Storage Time on Seminal Plasma Cytokine Contents

Analyses of SP samples are usually performed in large series for technical reasons, using commercial kits with 96 sample wells, for example. These samples usually remain frozen at either −20 °C or −80 °C for various intervals, depending on many variables, including experimental design, diagnostic strategies, etc. Therefore, this experiment aimed to assess the usefulness of these two freezing temperatures for long-term storage of SP samples for cytokine measurement. Individual SP from 20 ejaculates (one per boar) harvested immediately after ejaculation were used. Each SP sample was divided into three aliquots, with one remaining fresh for immediate cytokine measurement (baseline samples) and the other two frozen, one at −20 °C (Zanussi Tropic System) and the other at −80 °C (Ultra-Low Freezer). These two samples remained frozen for two months. The samples were thawed at RT for cytokine measurement.

### 2.4. Statistical Analysis

The SPSS v24.0 statistical package from IBM (IBM, Madrid, Spain) was used for statistical analysis. The Shapiro–Wilk test of the studentized residual data showed that the cytokine concentrations were not normally distributed. Therefore, the differences between baseline measurements and those of the experimental samples were analyzed using nonparametric tests, specifically, the Friedman test in Experiment 1, the Friedman test and the Wilcoxon signed-rank test in Experiment 2, and the Wilcoxon signed-rank test in Experiment 3. The results were expressed as median and percentiles. Differences were considered significant at *p* < 0.05. The concordance correlation coefficient (CCC) by Lin [32] was calculated to assess the agreement between the cytokine concentrations of baseline samples and those measured in the experimental samples of each experiment. The CCC values ranged from 0 to 1, with values less than 0.31 indicative of bad agreement, between 0.31 and 0.50 of poor agreement, between 0.51 and 0.70 of moderate agreement, between 0.71 and 0.90 of good agreement and greater than 0.90 indicative of very good agreement [33]. The Spearman’s Rho test was used to assess the linearity between the cytokine concentrations of baseline samples and those measured in the experimental samples of each experiment. A correlation coefficient (R value) greater than 0.70 indicated strong linearity [34].

## 3. Results

### 3.1. Experiment 1: Interval Ejaculation–Seminal Plasma Harvesting

**Figure 1:**
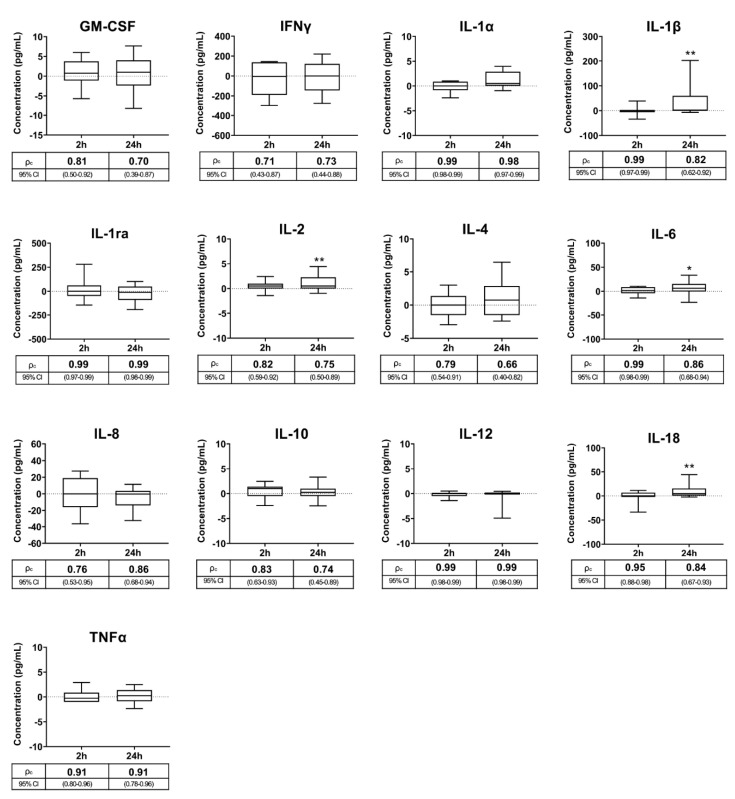
Box-and-whisker plot (horizontal line: median; box: 25/75 percentile; whisker: 10/90 percentile) showing the differences in cytokine concentrations measured in pig seminal plasma samples (SP) harvested 2 h and 24 h after ejaculate collection with respect to those harvested immediately after ejaculation (baseline samples, point 0 on Y axis). Below the X-axis is the concordance correlation coefficient (ρc) and 95% confidence interval (CI) showing agreement between the cytokine concentrations of the baseline and the experimental SP samples. Cytokines: Granulocyte macrophage colony-stimulating factor (GM-CSF), interferon-gamma (IFNγ), interleukin (IL)-1α, IL-1β, IL-1ra, IL-2, IL-4, IL-6, IL-8, IL-10, IL-12, IL-18 and tumor necrosis factor-α (TNFα). Asterisks indicate statistical differences with baseline data. ** *p* < 0.01; * *p* < 0.05.

Cytokine concentrations in SP samples harvested immediately after ejaculate collection (baseline values, Table 1) did not differ from those found after storing the collected semen samples at 17 °C for 2 h. In contrast, delaying SP harvesting by 24 h influenced (*p* < 0.05) SP concentrations of four of the 13 cytokines (Figure 1). Specifically, the concentrations of IL-1β, IL-2, IL-6 and IL-18 were 43.22%, 40.79%, 21.47% and 20.55% higher, respectively, in the SP samples harvested at 24 h compared to those harvested immediately after ejaculation. The cytokine concentrations measured in the SP samples harvested at 2 and 24 h after ejaculate collection showed good or very good agreement with those measured in SP samples harvested immediately after ejaculate collection (Figure 1). The cytokine concentrations measured in the SP samples harvested at 2 and 24 h after ejaculate collection showed good linearity (*p* < 0.01) with those measured in the SP samples harvested immediately after ejaculate collection (Table 2).

### 3.2. Experiment 2: Storage and Transport Conditions for Seminal Plasma before Analysis

**Figure 2:**
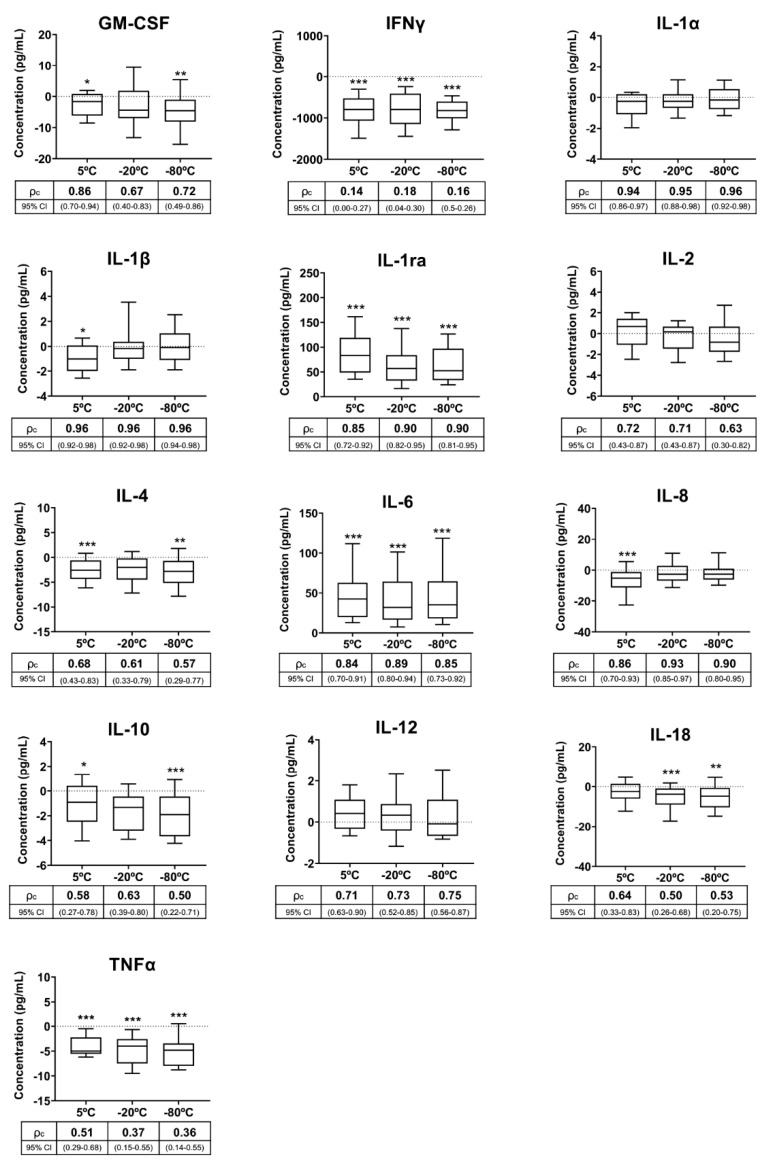
Box-and-whisker plot (horizontal line: median; box: 25/75 percentile; whisker: 10/90 percentile) showing the differences in cytokine concentrations measured in pig seminal plasma samples (SP) stored at 5 °C, −20 °C and −80 °C for 72 h with respect to those measured in fresh samples (baseline samples, point 0 in Y axis). Below the X-axis is the concordance correlation coefficient (ρc) and 95% confidence interval (CI), showing agreement between cytokine concentrations of baseline and experimental SP samples. Cytokines: Granulocyte macrophage colony-stimulating factor (GM-CSF), interferon-gamma (IFNγ), interleukin (IL)-1α, IL-1β, IL-1ra, IL-2, IL-4, IL-6, IL-8, IL-10, IL-12, IL-18 and tumor necrosis factor-α (TNFα). Asterisks indicate statistical differences with baseline data. *** *p* < 0.001; ** *p* < 0.01; * *p* < 0.05.

Cytokine concentrations were quantified in fresh and SP samples stored for 72 h at 5 °C, −20 °C and −80 °C. Table 3 shows the cytokine concentrations in the fresh samples, which were considered to be the baseline values. Ten of the 13 cytokines measured in SP samples were influenced (*p* < 0.05) by some of the storage conditions, specifically GM-CSF, IFNγ, IL-1β, IL-1ra, IL-4, IL-6, IL-8, IL-10, IL-18 and TNFα (Figure 2). The SP concentrations of eight of these ten cytokines were lower and those of the other two (IL-1ra and IL-6) were higher in stored SP samples compared to fresh SP samples. Storage at 5 °C influenced the concentration of nine cytokines, while freezing at −20 °C and −80 °C influenced the SP concentration of five and eight cytokines, respectively (Figure 2). The cytokines statistically influenced by the three storage conditions were IFNγ, IL-1ra, IL-6 and TNFα, which showed mean percentage variations above 20% in all storage conditions. The agreement between the concentrations measured in the fresh SP samples and those measured in the SP samples stored at 5 °C, −20 °C and −80 °C differed between cytokines and also between storage conditions (Figure 2). Agreement was good or very good for IL-1α, IL-1β, IL-1ra, IL-6, IL-8 and IL-12, regardless of storage condition. In contrast, agreement was bad for IFNγ, poor for TNFα and moderate for IL-4 and IL-10, regardless of storage conditions. The agreement for GM-CSF and IL-18 differed depending on storage conditions, with the best agreement demonstrated between fresh sample and those stored at 5 °C. The cytokine concentrations measured in the SP samples stored at 5 °C, −20 °C and −80 °C showed good linearity (*p* < 0.01) with those measured in the fresh SP samples (Table 2).

### 3.3. Experiment 3: Effects of Frozen Storage Time on Seminal Plasma Cytokine Contents

**Figure 3:**
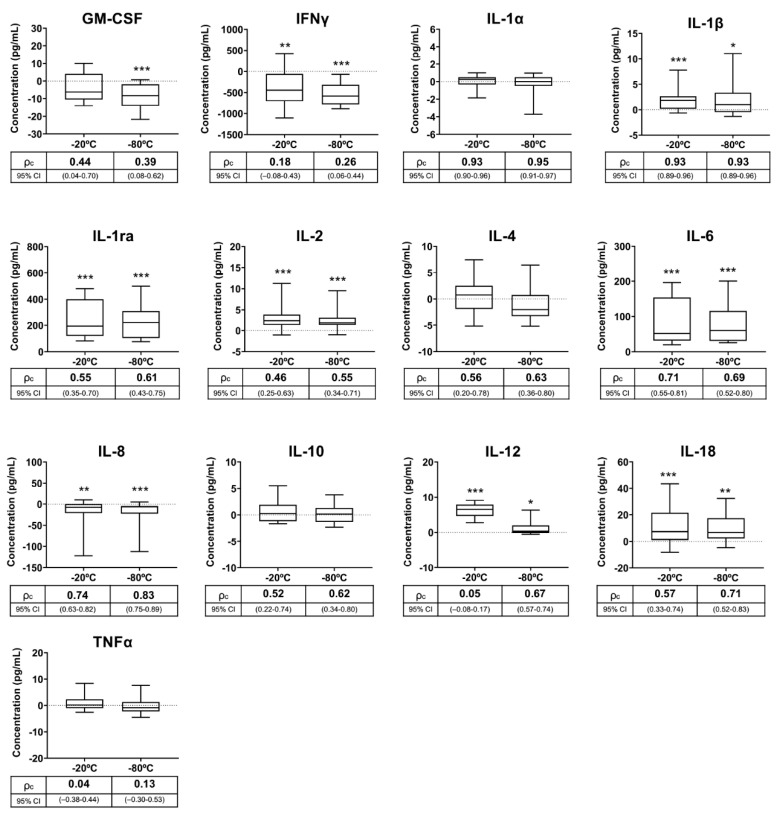
Box-and-whisker plot (horizontal line: median; box: 25/75 percentile; whisker: 10/90 percentile) showing differences in cytokine concentrations measured in pig seminal plasma samples (SP) stored at −20 °C and −80 °C for two months with respect to those measured in fresh samples (baseline samples, point 0 on the Y axis). Below the X-axis is the concordance correlation coefficient (ρc) and 95% confidence interval (CI), showing agreement between cytokine concentrations of the baseline and experimental SP samples. Cytokines: Granulocyte macrophage colony-stimulating factor (GM-CSF), interferon-gamma (IFNγ), interleukin (IL)-1α, IL-1β, IL-1ra, IL-2, IL-4, IL-6, IL-8, IL-10, IL-12, IL-18 and tumor necrosis factor-α (TNFα). Asterisks indicate statistical differences with baseline data. *** *p* < 0.001; ** *p* < 0.01; * *p* < 0.05.

Cytokines were measured in fresh and SP samples that remained stored at −20 °C and −80 °C for two months. Table 4 shows the cytokine baseline concentrations in fresh SP. The levels of nine of the 13 cytokines showed differences (*p* < 0.05) between fresh and stored samples, irrespective of the freezing storage temperature (Figure 3). The differences in the measured concentrations between the fresh and stored samples were above 25% in six of the nine cytokines, specifically in IFNγ, IL-1ra, IL-2, IL-6, IL-12 and IL-18. In four of these cytokines, the differences between samples that were fresh and stored at −20 °C were greater than those between samples that were fresh and stored at −80 °C. The agreement between the concentrations measured in the fresh SP samples and those measured in the SP samples stored for two months at −20 °C or at −80 °C differed between cytokines and also between storage conditions (Figure 3). Agreement was good or very good for IL-1α, IL-1β, IL-6 and IL-8, regardless of storage conditions. In contrast, agreement was bad for IFNγ and TNFα, poor for GM-CSF and moderate for IL-1ra and IL-10, regardless of storage conditions. Agreement for IL-2, IL-4, IL-12 and IL-18 differed according to storage conditions, but was better between fresh and −80 °C stored samples. The linearity between fresh and stored SP samples was poor or moderate for GM-CSF, IFNγ, IL-10 and TNFα, regardless of storage conditions. IL-2, IL-4, IL-12 and IL-18 concentrations of the SP samples stored at −20 °C also showed poor or moderate linearity with those of the fresh SP samples (Table 2).

## 4. Discussion

To the best of our knowledge, this study was the first to evaluate how realistic procedures for both semen handling and SP storage influence measurable SP cytokine levels. Although the focus was on breeding boars delivering semen for the production of doses for artificial insemination (AI), particularly considering the domination of AI in commercial pig breeding worldwide, the results demonstrate comparative value to other species, including humans. The results are indeed intended to be a useful reference for clinicians or researchers regarding the best practices for semen handling and SP storage to achieve reliable and comparable results of SP concentrations of the cytokine/s of interest.

The battery of cytokines was measured using the Luminex bead array assay, which is currently the procedure of choice for clinical cytokine analysis as it allows simultaneous measurement of several cytokines and has a good well-to-well and day-to-day reproducibility [35,36]. However, these antibody kits may show variability between production batches, particularly at detection limits, thereby hindering the comparison of results between assays [37]. For the trials conducted in the present study, we used single-batch kits and identical standard curve ranges, so the results achieved in the different trials were fully comparable. Taken together, the results highlighted the existence of wide ranges in SP concentrations for many cytokines, which was also evident among experiments, particularly for some cytokines, because the boar semen donors were different. This may have been caused by the clear differences observed between ejaculates/males in the measured SP concentrations, consistent with previous studies that highlighted clear variability between male breeding pigs in the concentrations of SP cytokines. [38,39,40]. Similar high variability in cytokine concentrations was also observed in the SP of healthy men [30,31].

The present study included three separate experiments, with the first one evaluating how a not-unusual delay in SP harvesting, often due to workload-related problems or a lack of technical resources, influences the measurable SP concentrations of cytokines. Although the concentrations of some cytokines differed from those of the 0 h SP samples, a delay of 2 h or even 24 h in the SP harvesting did not show a determining impact on the analyzed SP cytokine concentrations. There was good or very good agreement between the measurements of the SP samples collected at 0 h and those collected either at 2 h or 24 h after ejaculate collection. These SP harvesting intervals would allow operators to collect semen without jeopardizing working routines at AI centers or affecting SP cytokine levels. Moreover, delaying SP harvesting up to 24 h would allow raw semen samples to be sent from ejaculate collection centers to reference laboratories for the measurement of SP cytokines, a practice sometimes necessary due to the technical limitations of some livestock ejaculate collection centers (for example, lack of centrifuges), together with the distance to clinical laboratories. Of further relevance, semen and blood clearly differ, considering that a delay of less than 2 h in blood plasma collection caused increases in plasma cytokine levels, as demonstrated in humans [41]; therefore, the collection of blood plasma or serum as soon as possible after blood draw is recommended [42], which would not be necessary for SP, at least in pigs. The increase in the levels of some cytokines in the SP samples harvested 24 h after ejaculate collection, specifically in IL-1β, IL-2 and Il-18, may have been caused by the release of certain amounts of these cytokines from the sperm into the SP. However, this increase did not appear to be a relevant barrier to reliable measurement of SP cytokines. It should be noted that the semen of livestock species, particularly those selected and kept for production of AI semen doses, usually contains low proportions of other cells besides spermatozoa [43]. However, they always contain cytokines in SP, most notoriously when spermatozoa lose membrane integrity [44]. Sperm membrane disruption surely occurs within semen samples stored for 24 h, since ejaculated spermatozoa, even when properly extended, gradually lose integrity of the membrane over time [45,46].

Ideally, cytokines should be measured in fresh samples to identify true baseline levels. However, this practice is unusual for semen since, as mentioned above, livestock ejaculate collection centers are far from reference clinical laboratories, thereby requiring shipment and a consequent delay in analysis. Furthermore, SP samples may be stored for a short period of time before analysis once they reach clinical laboratories. Considering these scenarios, the second experiment evaluated the stability of cytokines in SP samples handled under three realistic storage and/or shipping conditions, namely cooling at 5 °C or freezing to −20 °C or −80 °C for 72 h. Not all cytokines behaved in the same way in the stored samples. While some showed good or very good agreement with the fresh sample measurements, others showed bad or poor agreement. The instability of cytokines in stored SP samples, irrespective of storage temperature, were expected to some extent, since similar results were achieved in stored human blood plasma samples [47]. It should be noted that the SP concentrations of many affected cytokines varied, as expected, following the trends of the three storage conditions tested (increasing or decreasing), but the magnitude of variation between storage conditions clearly differed among cytokines, as showed by the CCC. This inconsistency between cytokines for measurable concentrations after storage of SP samples was also shown in human blood plasma samples [21,23,24,48]. This instability was particularly evident for IFNγ, TNFα, IL-4 and IL-10, which showed decreased concentrations in stored SP samples relative to fresh SP samples irrespective of storage temperature. Two main causes were discussed to explain why the levels of many cytokines differ between fresh and stored blood plasma samples [23,48,49]. Specifically, instability during storage could lead to degradation and, therefore, decreased measurable levels, while structure modification during storage could lead to either decreased or increased measurable levels among stored samples. This instability is one of the major causes affecting the reliable measurement of cytokine levels in body fluids and is particularly tangible in stored samples, as many are temperature labile [47,50]. Therefore, a decrease in measurable levels of some cytokines, as detected in the stored pig SP samples, is expected. Despite the good agreement between the fresh and stored SP sample IL-1ra and IL-6 measurements, increased concentrations of these cytokines in the stored samples was surprising. The increase in cytokine levels during short-term storage, either by refrigeration or freezing, was also demonstrated in human blood plasma, but no reasons explaining this phenomenon were given [42]. Modifications in cytokine structure during storage could be a plausible explanation of this increase, as molecular structure changes encompass the other main reason to explain the temperature instability of cytokines [47]. In this regard, proteins are known to undergo a phenomenon known as cold denaturation, leading to structural changes [51] and facilitating the binding of more antibodies, thereby leading to false higher quantity measurements [52]. More studies are needed to test this hypothesis.

In the third and last experiment, the influence of a relatively long-term freeze storage on measurable levels of seminal cytokines was evaluated. Checking the stability of cytokines in SP samples stored for a relatively long time may be relevant to the work dynamics of andrology laboratories. It is widely accepted that body fluids must be kept frozen to preserve their biomolecules in the long-term [53]. Therefore, SP samples were stored at −20 °C and −80 °C for two months, with the results showing that many of the measured cytokines were unable to remain stable over time in the frozen samples, regardless of whether they were stored at −20 °C or at −80 °C. These results were expected looking at those achieved in the previous experiment. Noticeably, many unstable cytokines exhibited higher concentrations in the stored SP samples than in the fresh SP samples. Therefore, it could be argued that long-term freeze storage causes structural changes in many cytokines, thereby altering their quantification, at least when using the Luminex bead array assay.

Taken together, the findings from the second and third experiments highlighted that none of the storage conditions tested are ideal for preserving the original levels of the cytokines of interest in the SP samples, at least not for those quantified in this study. These results limit the practical clinical use of seminal cytokine concentrations as biomarkers of male reproductive health. This study also explored whether the measured cytokine concentrations in fresh SP samples and stored SP samples follow a linear relationship. With few exceptions, the linearity was good for both short- and long-term storage conditions. This good linearity suggests that the storage conditions tested may be useful, provided that they are chosen based on consensus and widely accepted by clinical laboratories. Only in this way would it be possible to correctly compare results between different clinical trials, since the cut-off concentrations between the physiological and pathological reproductive conditions may differ between storage conditions. Before deciding which condition for SP storage is better, it is important to consider that some cytokines are at very low measurable levels in the SP of healthy boars, as evidenced in this study and others [38,39,40]. Therefore, the conditions chosen should be those that guarantee the highest measurable levels for the cytokines of interest. According to the linearity results, short-term storage, irrespective of temperature, is better than long-term storage, and −80 °C is better than −20 °C when long-term storage is indispensable.

## 5. Conclusions

In summary, the results showed that delaying SP harvesting by up to 24 h after ejaculate collection does not have a substantial impact on SP cytokine measurements. In contrast, there are no ideal short- or long-term storage conditions for SP samples that allow for reliable measurement of the original levels of all cytokines of interest, at least not among those quantified in this study. Therefore, measurements should be made on fresh SP samples whenever possible. Cytokine instability varied substantially between the tested storage conditions. More cytokines were more unstable in the SP samples stored for a long time than those for a short time. While IL-1α, IL-1β, IL-6 and IL-8 remained quite stable under all tested storage conditions, others, such as IFNγ and TNFα, showed instability throughout. Consequently, SP storage protocols should be specifically adapted for each cytokine to be measured. Beyond its practical utility, the results of this study are a clear wake-up call regarding the need for consensus between laboratories on the conditions used for semen handling and SP storage for cytokine measurements. This consensus is required for reliable results during clinical diagnosis and comparisons between clinical trials or laboratories. If there is consensus, the storage conditions tested could be considered for SP cytokine measurements, since the linearity between measurements in fresh and stored SP samples was high for most of the analyzed cytokines.

## Figures and Tables

**Table 1:** Experiment 1: Cytokine concentrations in pig fresh seminal plasma samples (baseline samples, *n* = 20). Cytokines: Granulocyte macrophage colony-stimulating factor (GM-CSF), interferon-gamma (IFNγ), interleukin (IL)-1α, IL-1β, IL-1ra, IL-2, IL-4, IL-6, IL-8, IL-10, IL-12, IL-18 and tumor necrosis factor-α (TNFα).

Cytokine (pg/mL)	Median (25th, 75th Percentiles)	Range
GM-CSF	31.00 (27.50, 34.88)	27.50
IFNγ	1317.00 (1073, 1496)	667.50
IL-1α	10.75 (4.00, 25.38)	37.50
IL-1β	18.75 (15.63, 99.50)	480.50
IL-1ra	626.30 (293.90, 1763.00)	3621.00
IL-2	5.25 (3.62, 9.00)	11.00
IL-4	21.75 (18.63, 23.50)	9.50
IL-6	83.25 (64.25, 151.90)	347.50
IL-8	32.75 (22.50, 54.88)	99.80
IL-10	9.50 (8.62, 13.63)	10.50
IL-12	3.00 (3.00, 4.25)	68.20
IL-18	20.50 (4.25, 77.63)	195.00
TNFα	18.25 (13.75, 23.50)	19.00

**Table 2:** Spearman’s correlation coefficients (significance in brackets) showing linearity between the cytokine concentrations measured in baseline seminal plasma (SP) samples and those measured in experimental SP samples for each experiment. Cytokines: Granulocyte macrophage colony-stimulating factor (GM-CSF), interferon-gamma (IFNγ), interleukin (IL)-1α, IL-1β, IL-1ra, IL-2, IL-4, IL-6, IL-8, IL-10, IL-12, IL-18 and tumor necrosis factor-α (TNFα). Experiment 1: SP samples harvested immediately (0 h), 2 h and 24 h after ejaculation; Experiment 2: Fresh and stored SP samples for 72 h at 5 °C, −20 °C and −80 °C; Experiment 3: Fresh and stored SP samples for two months at −20 °C and −80 °C.

Cytokine	Spearman Correlation (*p*-Value)						
	Experiment 1		Experiment 2			Experiment 3	
	2 h vs. 0 h	24 h vs. 0 h	5 °C vs. Fresh	−20 °C vs. Fresh	−80 °C vs. Fresh	−20 °C vs. Fresh	−80 °C vs. Fresh
GM-CSF	0.83 (<0.001)	0.70 (0.001)	0.89 (<0.001)	0.74 (<0.001)	0.81 (<0.001)	0.48 (0.032)	0.56 (0.010)
IFNγ	0.62 (0.004)	0.74 (0.002)	0.79 (<0.001)	0.56 (ns)	0.72 (0.006)	0.32 (ns)	0.62 (0.003)
IL-1α	0.98 (<0.001)	0.99 (<0.00)	0.90 (<0.001)	0.90 (<0.001)	0.88 (<0.001)	0.81 (<0.001)	0.85 (<0.001)
IL-1β	0.86 (<0.001)	0.81 (<0.00)	0.92 (<0.001)	0.93 (<0.001)	0.95 (<0.001)	0.93 (<0.001)	0.85 (<0.001)
IL-1ra	0.97 (<0.001)	0.99 (<0.00)	0.96 (<0.001)	0.96 (<0.001)	0.97 (<0.001)	0.94 (<0.001)	0.96 (<0.001)
IL-2	0.88 (<0.001)	0.89 (<0.00)	0.70 (<0.001)	0.71 (0.006)	0.70 (0.003)	0.57 (0.008)	0.83 (<0.001)
IL-4	0.79 (<0.001)	0.76 (<0.00)	0.81 (<0.001)	0.73 (0.011)	0.70 (<0.011)	0.60 (0.005)	0.72 (<0.001)
IL-6	0.95 (<0.001)	0.91 (<0.00)	0.96 (<0.001)	0.97 (<0.001)	0.96 (<0.001)	0.98 (<0.001)	0.97 (<0.001)
IL-8	0.68 (<0.001)	0.75 (<0.00)	0.83 (<0.001)	0.91 (<0.001)	0.94 (<0.001)	0.77 (<0.001)	0.86 (<0.001)
IL-10	0.82 (<0.001)	0.84 (<0.00)	0.66 (0.003)	0.84 (0.010)	0.71 (<0.010)	0.53 (0.016)	0.65 (0.002)
IL-12	0.70 (<0.001)	0.90 (<0.00)	0.86 (<0.001)	0.84 (<0.001)	0.83 (<0.001)	0.34 (ns)	0.84 (<0.001)
IL-18	0.95 (<0.001)	0.93 (<0.00)	0.82 (<0.001)	0.78 (<0.001)	0.80 (<0.001)	0.63 (0.003)	0.86 (<0.001)
TNFα	0.92 (<0.001)	0.93 (<0.00)	0.83 (<0.001)	0.71 (0.009)	0.72 (<0.001)	0.40 (ns)	0.60 (0.005)

**Table 3:** Experiment 2: Cytokine concentrations in pig fresh seminal plasma samples (baseline samples, *n* = 22). Cytokines: Granulocyte macrophage colony-stimulating factor (GM-CSF), interferon-gamma (IFNγ), interleukin (IL)-1α, IL-1β, IL-1ra, IL-2, IL-4, IL-6, IL-8, IL-10, IL-12, IL-18 and tumor necrosis factor-α (TNFα).

Cytokine (pg/mL)	Median (25th, 75th Percentiles)	Range
GM-CSF	44.34 (40.17, 54.42)	34.66
IFNγ	2457.00 (2227.00, 2699.00)	1223.00
IL-1α	4.83 (4.16, 7.08)	12.00
IL-1β	16.17 (14.83, 19.58)	19.67
IL-1ra	147.80 (57.42, 288,3)	449.00
IL-2	10.33 (7.58, 11.17)	7.00
IL-4	32.67 (29.34, 34.75)	15.67
IL-6	82.17 (47.58, 225.20)	290.00
IL-8	34.00 (29.50, 53.50)	63.33
IL-10	13.67 (12.00, 14.42)	7.67
IL-12	6.50 (4.91, 7.00)	4.34
IL-18	15.34 (9.25, 27.00)	32.67
TNFα	23.67 (20.33, 25.00)	11.33

**Table 4:** Experiment 3: Cytokine concentrations in pig fresh seminal plasma samples (baseline samples, *n* = 20). Cytokines: Granulocyte macrophage colony-stimulating factor (GM-CSF), interferon-gamma (IFNγ), interleukin (IL)-1α, IL-1β, IL-1ra, IL-2, IL-4, IL-6, IL-8, IL-10, IL-12, IL-18 and tumor necrosis factor-α (TNFα).

Cytokine (pg/mL)	Median (25th, 75th Percentiles)	Range
GM-CSF	39.67 (36.67, 47.33)	42.33
IFNγ	2004.00 (1881.00, 2219.00)	1097
IL-1α	4.00 (4.00, 5.75)	26.67
IL-1β	17.33 (16.00, 22.00)	38.33
IL-1ra	138.00 (51.25, 225.20)	757.00
IL-2	7.67 (6.00, 8.00)	13.00
IL-4	29.33 (27.00, 31.67)	14.00
IL-6	77.17 (40.33, 209.10)	278.70
IL-8	37.17 (25.83, 56.67)	342.00
IL-10	11.67 (10.50, 12.58)	9.67
IL-12	5.00 (4.08, 5.67)	14.33
IL-18	15.33 (10.08, 27.00)	62.67
TNFα	20.50 (18.67, 21.59)	32.34
